# A nomogram incorporating nutritional risk for predicting 31-day unplanned readmission in adults with type 2 diabetes mellitus using LASSO regression

**DOI:** 10.3389/fnut.2026.1863130

**Published:** 2026-09-30

**Authors:** Jing Cao, Qiumei Shi, Zikai Yu, Hebin Xie

**Affiliations:** The Affiliated Changsha Central Hospital, Hengyang Medical School, University of South China, Changsha, China

**Keywords:** LASSO regression, nomogram, nutritional risk, type 2 diabetes mellitus, unplanned readmission within 31 days

## Abstract

**Objective:**

To develop and validate a nomogram incorporating nutritional risk for predicting 31-day unplanned readmission in patients with type 2 diabetes mellitus (T2DM).

**Methods:**

This retrospective study included 1,627 T2DM patients. A nomogram was constructed using least absolute shrinkage and selection operator (LASSO) regression on a training set (*n* = 1,139) and an internal hold-out set (*n* = 488). Predictors were selected from demographics, lifestyle, clinical, and nutritional parameters. Model performance was evaluated using ROC curves, calibration plots, and decision curve analysis.

**Results:**

The 31-day unplanned readmission rate was 18.50%. Six predictors were identified: smoking history, peripheral vascular disease in patients with type 2 diabetes mellitus (PVD in T2DM), diabetes duration, glycated hemoglobin (HbA1c), nutritional risk and neutrophil-to-lymphocyte ratio (NLR). The nomogram showed good discrimination in both training (AUC = 0.846; 95% CI: 0.814–0.878) and internal hold-out set (AUC = 0.817; 95% CI: 0.761–0.873), with acceptable calibration.

**Conclusion:**

In this single-center retrospective cohort, the nomogram demonstrated potential for predicting 31-day unplanned readmission in T2DM patients by integrating clinical, metabolic, inflammatory, and nutritional dimensions. Routine nutritional assessment at admission may help identify high-risk individuals; however, external validation in geographically and temporally diverse populations is necessary before clinical application.

## Introduction

The prevalence of diabetes worldwide is on a continuous upward trend and is estimated to reach around 800 million by 2045 (1). China is one of the countries with the largest increase in the prevalence of diabetes and the highest mortality rate in the world. Among them, patients with Type 2 diabetes mellitus (T2DM) account for 90.0% of all diabetes patients (2). Patients with type 2 diabetes have insulin resistance and/or insufficient insulin secretion in B-cells, which prevents glucose from effectively entering cells for utilization. Long-term hyperglycemia can cause diabetic complications such as kidney, eye and nerve problems (3, 4), aggravating the disease and physical and mental burden. In 2024, global healthcare expenses related to diabetes exceeded 1 trillion US dollars for the first time, and China’s medical expenses for diabetes accounted for 16.6% of the global total expenditure (5).

Patients with type 2 diabetes commonly have nutritional issues. A meta-analysis indicates that the prevalence of malnutrition among diabetes patients is 33%, and the proportion of those with nutritional risks is as high as 44% (6). Moreover, multiple micronutrient deficiencies are relatively common among patients with type 2 diabetes, affecting 45.3% of them. The most common ones are vitamin D (60.45%) and magnesium (41.95%) (7). These nutritional disorders are associated with poor clinical outcomes, including increased risk of hospitalization and rehospitalization.

Patients with diabetes frequently require hospitalization because of related complications. In 2017, one sixth of the hospital beds in the UK were occupied by diabetic patients, and it is expected that this proportion will rise to one quarter by 2030 (8). Timple et al. (9) found through a retrospective analysis of the medical records of 400 elderly patients with type 2 diabetes in a hospital in Southern California, USA, that the readmission rate within 30 days after discharge was 15.3%. A retrospective study by Regassa et al. (10) on 458 patients with type 2 diabetes in Ethiopia showed that the readmission rate within 30 days after discharge was 29.5%. A retrospective study in China (11) showed that the readmission rate within 30 days after discharge for diabetic patients in a certain hospital in Haikou City was 22.9%. Readmission imposes a huge disease burden and economic pressure on diabetic patients. Reducing the readmission rate of patients can significantly lower healthcare costs (12).

Notably, malnutrition has been identified as a strong independent predictor of adverse short-term outcomes, including hospital readmission, in patients with chronic diseases. However, nutritional risk screening is not routinely performed for hospitalized T2DM patients, partly due to a lack of consensus on screening tools and underestimation of its clinical relevance. Existing prediction models for readmission in diabetic patients are not only limited in number but also often rely on administrative data or single clinical parameters, whereas models that specifically incorporate nutritional risk indicators for unplanned readmission are even rarer. Readmission is a widely used indicator for evaluating medical quality internationally. In 2011, China also included 31-day unplanned readmission as a quality management and control target for hospitals (13). Therefore, this retrospective study aimed to develop a prediction model for 31-day unplanned readmission in adult T2DM patients by integration of nutritional, immune, and endocrine indicators using least absolute shrinkage and selection operator (LASSO) regression, with the goal of facilitating early identification and targeted intervention.

## Method

### Research design and research subjects

This retrospective study included patients with type 2 diabetes who were hospitalized at Changsha Central Hospital from January 2024 to December 2024. The inclusion criteria were: ① diagnosed with T2DM according to the Chinese Diabetes Prevention and Treatment Guidelines (2024 edition) (14); ② aged ≥18 years; ③ complete data records. Exclusion criteria: patients whose readmission was not due to T2DM or its related complications, planned readmission, missing medical record information, pregnancy, malignant tumor, or incomplete data records.

The study has been approved by the Human Ethics Review Committee of Changsha Central Hospital, and the study adhered to the principles of the Declaration of Helsinki (Ethical approval number: Medical Review No. 126 of 2026).

### Data collection

Data of eligible patients were extracted from the electronic medical record system, laboratory information system, and mobile nursing system. Collected information included sociodemographic characteristics, lifestyle and behavioral factors (e.g., smoking and drinking), nutritional risk assessed using the Nutritional Risk Screening 2002 (NRS 2002) tool at admission, and relevant clinical data.

Readmission events were identified retrospectively from two complementary sources: (1) the readmission statistics table of our hospital’s electronic medical record (EMR) system (capturing readmissions to our hospital); and (2) routine telephone follow-up records by nurses within 1 month after discharge as part of standard clinical care (capturing readmissions to other hospitals). Two specialist physicians independently reviewed all potential readmission events, determined whether each was unplanned and related to the previous admission, and if disagreements arose, they were resolved by discussion with a senior attending physician. Inter-rater agreement was *k* = 0.92 (95% CI: 0.87–0.97).

### Missing data handling

After excluding 123 patients who did not meet the inclusion criteria and 78 patients with missing data on the outcome or any candidate predictor, a complete-case dataset of 1,627 patients was obtained (Figure 1). The proportion of missing data was low (4.3% of the initial cohort).

**Figure 1:**
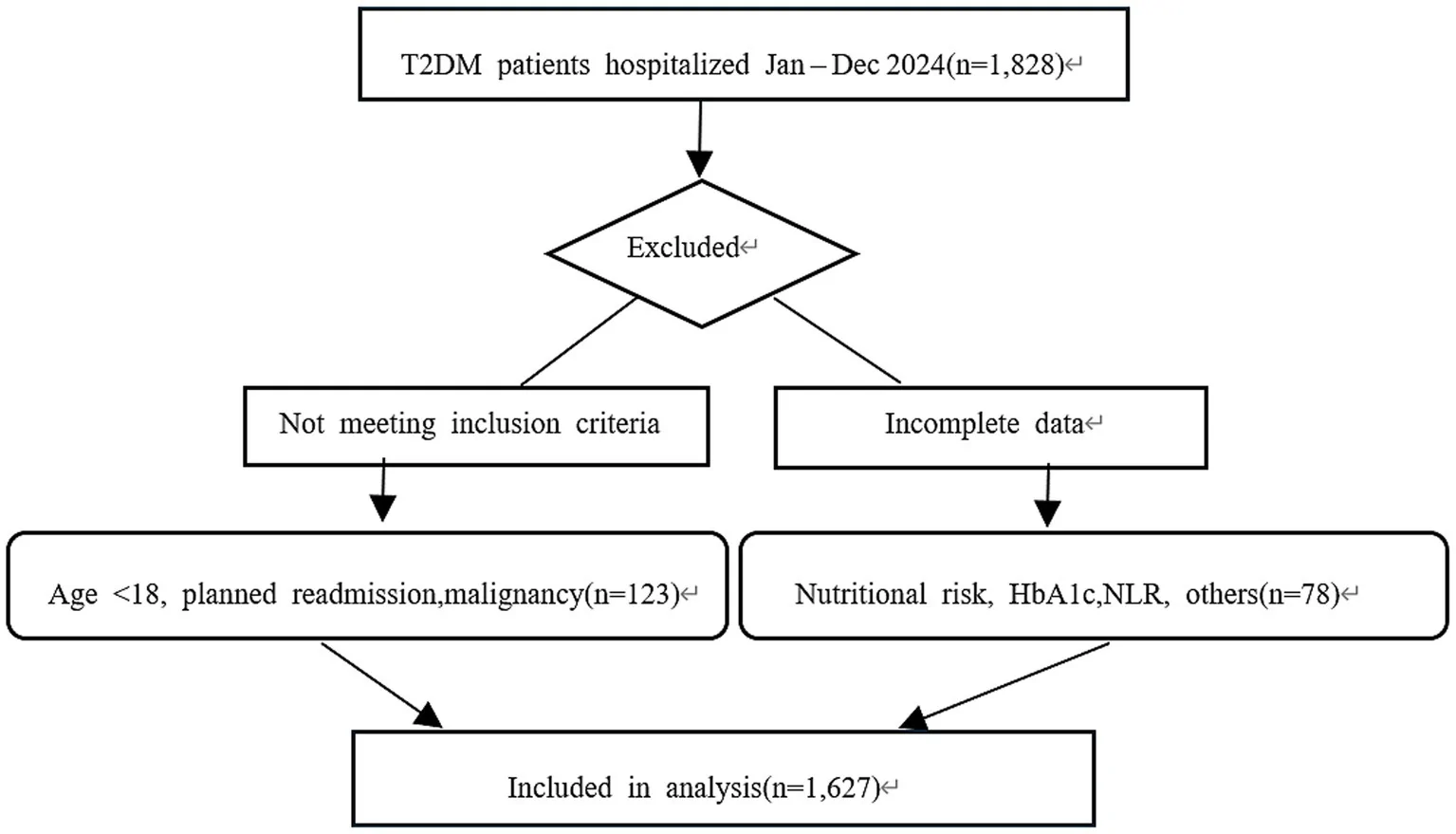
Flow diagram of patient selection.

### Unplanned readmission within 31 days

Unplanned readmission refers to the situation where a patient is readmitted within 31 days after discharge from the previous treatment (an unplanned readmission), and the cause of readmission is the same as or related to the previous hospitalization (15). The 31-day window follows the Chinese national hospital quality management indicator (National Health Commission of China, 2011) (13). The primary endpoint was unplanned readmission within 31 days. For patients with multiple readmission within 31 days after discharge, only the first readmission was recorded.

### Nutritional risk screening

Nutritional risk was assessed at admission using the NRS 2002 tool (16). This screening instrument evaluates a patient’s nutritional status, disease severity, and age. A total score of ≥ 3 points was defined as indicative of nutritional risk, based on the original validation study (17). All screenings were performed by trained nurses within 24 h of admission. To ensure consistency, the same nurse conducted the screening for all patients.

### Statistical analysis

Statistical analyses were performed using R software (version 4.3.2) and STATA (version 13.1; STATA Corp., College Station, TX). Normally distributed data were presented as mean ± tandard deviation and compared using the Student’s *t*-test. Non-normally distributed variables were expressed as median (25th percentile, 75th percentile) and compared using the Mann–Whitney *U* test. Categorical data were presented as numbers (percentages) and compared using the Pearson chi-square test or Fisher’s exact test. Using set.seed (123) to set a random seed, the total sample was randomly divided into a training set and an internal hold-out set at a ratio of 7:3. No external validation was performed. First, LASSO regression was applied to all 36 candidate predictors (Supplementary Table S7) using the glmnet package (version 4.1.8) in R. The cv.glmnet function with 10-fold cross-validation was used to select the penalty parameter λ, which was chosen according to the one-standard-error rule (λ₁se). Continuous predictors were automatically standardized (default standardize = TRUE in glmnet). Subsequently, the selected variables were refitted into a multivariate logistic regression model using maximum-likelihood estimation to obtain regression coefficients and odds ratios, and a nomogram was developed based on this refitted model. The discriminative ability of the model was evaluated using the receiver operating characteristic (ROC) curve. Bootstrap resampling (*B* = 1,000 repetitions) was applied only to the training set to obtain optimism-corrected performance metrics. The internal hold-out set was not involved in the bootstrap procedure and was used exclusively for the final evaluation of the refitted model. Calibration curves were plotted to visually assess the agreement between the predicted probabilities and the actual observed frequencies, with the Hosmer-Lemeshow goodness-of-fit test serving as a supplementary assessment. Decision curve analysis (DCA) was applied to evaluate the clinical utility of the model. A two-sided *p*-value < 0.05 was considered statistically significant. The R code used for model development and validation is available from the corresponding author upon reasonable request.

## Result

### Unplanned readmission rate

A total of 1,627 patients with type 2 diabetes were included in the final analysis. Among these, 301 (18.50%) experienced an unplanned readmission within 31 days after discharge. A random seed was set, and the overall sample was randomly divided into a training set (*n* = 1,139) and an internal hold-out set (*n* = 488) at a ratio of 7:3.

### The characteristics of the two groups of patients in the training set

There were statistically significant differences in occupation, smoking, diabetic nephropathy in patients with type 2 diabetes mellitus (DN in T2DM), diabetic retinopathy in patients with type 2 diabetes mellitus (DR in T2DM), peripheral vascular disease in patients with type 2 diabetes mellitus (PVD in T2DM), nutritional risk, duration of diabetes, glycated hemoglobin (HbA1c) and neutrophil-to-lymphocyte ratio (NLR) between the two groups of patients (*p* < 0.05), and the differences are shown in Table 1.

**Table 1:** Comparison of general clinical data of the two groups of patients in the training set.

Variables	Classification	No unplanned readmission ≤ 31 day (*n* = 920)	Unplanned readmission≤31 day (*n* = 219)	*t*/*χ*^2^/*z*	*p*
Age, year (mean ± SD)		63.00 ± 11.04	63.15 ± 10.68	−0.173	0.863
Residences, *n* (%)	City	796 (86.52)	198 (90.41)	2.408	0.121
	Rural	124 (13.48)	21 (9.59)		
Education, *n* (%)	Junior high school and below	775 (84.24)	180 (82.19)	5.299	0.071
	High school/technical secondary school	72 (7.83)	12 (5.48)		
	University degree or above	73 (7.93)	27 (12.33)		
Occupation, *n* (%)	Workers	157 (17.07)	62 (28.31)	44.858	<0.001
	Staffs	307 (33.37)	40 (18.26)		
	Farmers	231 (25.11)	69 (31.51)		
	Civil servants	200 (21.74)	31 (14.16)		
	Others	25 (2.72)	17 (7.76)		
Smoking, *n* (%)	No	878 (95.43)	193 (88.13)	16.824	<0.001
	Yes	42 (4.57)	26 (11.87)		
Drinking, *n* (%)	No	894 (97.17)	210 (95.89)	0.978	0.323
	Yes	26 (2.83)	9 (4.11)		
Gender, *n* (%)	Male	519 (56.41)	130 (59.36)	0.627	0.428
	Female	401 (43.59)	89 (40.64)		
Marital status, *n* (%)	unmarried	24 (2.61)	8 (3.65)	0.712	0.701
	Married	874 (95.00)	206 (94.06)		
	Others	22 (2.39)	5 (2.28)		
Insurance type, *n* (%)	Medical insurance for urban workers	622 (67.61)	132 (60.27)	5.407	0.144
	Medical insurance for urban residents	226 (24.57)	70 (31.96)		
	New rural cooperative medical	51 (5.54)	11 (5.02)		
	Others	21 (2.28)	6 (2.74)		
Admission route, *n* (%)	Outpatient department	870 (94.57)	206 (94.06)	0.085	0.771
	Emergency department	50 (5.43)	13 (5.94)		
Discharge method, *n* (%)	Doctor’s order to leave the hospital	888 (96.52)	211 (96.35)	–	0.812^#^
	Discharge against medical advice	27 (2.93)	6 (2.74)		
	Transfer to another hospital	5 (0.54)	2 (0.91)		
Length of hospital stay, day, Median (p25, p75)		8.00 (7.00, 10.00)	9.00 (7.00, 11.00)	−1.510	0.131
Clinical pathway, *n* (%)	Not entered	249 (27.07)	60 (27.4)	–	0.083^#^
	Exited	6 (0.65)	5 (2.28)		
	Completed	665 (72.28)	154 (70.32)		
DN in T2DM, *n* (%)	No	690 (75.00)	141 (64.38)	10.106	0.001
	Yes	230 (25.00)	78 (35.62)		
DR in T2DM, *n* (%)	No	758 (82.39)	164 (74.89)	6.461	0.011
	Yes	162 (17.61)	55 (25.11)		
PVD in T2DM, *n* (%)	No	768 (83.48)	153 (69.86)	21.188	<0.001
	Yes	152 (16.52)	66 (30.14)		
Nutritional risk, *n* (%)	No	814 (88.48)	173 (79.00)	13.755	<0.001
	Yes	106 (11.52)	46 (21.00)		
Hypertension, *n* (%)	No	375 (40.76)	81 (36.99)	1.050	0.306
	Yes	545 (59.24)	138 (63.01)		
Coronary heart disease, *n* (%)	No	684 (74.35)	152 (69.41)	2.212	0.137
	Yes	236 (25.65)	67 (30.59)		
Atrial fibrillation, *n* (%)	No	896 (97.39)	213 (97.26)	0.012	0.913
	Yes	24 (2.61)	6 (2.74)		
Cerebral infarction, *n* (%)	No	814 (88.48)	195 (89.04)	0.055	0.814
	Yes	106 (11.52)	24 (10.96)		
Chronic obstructive pulmonary disease, *n* (%)	No	897 (97.50)	210 (95.89)	1.678	0.195
	Yes	23 (2.50)	9 (4.11)		
Hyperlipidemia, *n* (%)	No	447 (48.59)	105 (47.95)	0.029	0.864
	Yes	473 (51.41)	114 (52.05)		
Hypothyroidism, *n* (%)	No	891 (96.85)	210 (95.89)	0.503	0.478
	Yes	29 (3.15)	9 (4.11)		
Operationed, *n* (%)	No	768 (83.48)	185 (84.47)	0.129	0.720
	Yes	152 (16.52)	34 (15.53)		
Duration of diabetes, year, Median (p25, p75)		11.10 (6.00, 16.10)	13.40 (8.50, 17.80)	−4.103	<0.001
Lower extremity venous thrombosis, *n* (%)	No	630 (68.48)	137 (62.56)	2.820	0.093
	Yes	290 (31.52)	82 (37.44)		
HbA1c, %, (mean ± SD)		7.66 ± 2.49	8.10 ± 2.14	−2.387	0.017
FBG, mmol/L, (mean ± SD)		7.49 ± 3.29	7.96 ± 3.56	−1.897	0.058
DD, mg/L, Median (p25, p75)		1.20 (0.20, 1.20)	1.20 (0.30, 1.20)	−0.315	0.752
UA, μmol/L, Median (p25, p75)		272.22 (189.63, 350.95)	274.00 (217.00, 357.71)	−1.139	0.255
LDL, mmol/L, Median (p25, p75)		2.51 (2.23, 2.77)	2.51 (2.49, 2.83)	−1.560	0.119
TG, mmol/L, Median (p25, p75)		1.77 (1.24, 2.5)	1.77 (1.30, 2.51)	−0.552	0.581
HDL, mmol/L, Median (p25, p75)		1.14 (0.9, 1.31)	1.14 (0.92, 1.31)	−0.014	0.989
TC, mmol/L, Median (p25, p75)		4.41 (3.77, 5.20)	4.46 (3.84, 5.30)	−1.237	0.216
NLR, (mean ± SD)		3.21 ± 1.65	3.63 ± 1.170	−3.583	<0.001

^*^FBG, Fasting blood glucose; HbA1c, glycated hemoglobin, DD, D-Dimer; Scr, serum creatinine; UA, uric acid, LDL, Low-density lipoprotein; TG, Triglycerides; HDL, High-density lipoprotein; TC, Total cholesterol; NLR, Neutrophil-to-Lymphocyte Ratio; PVD in T2DM, Peripheral vascular disease in patient with type 2 diabetes; DN in T2DM, Type 2 Diabetic Nephropathy.

For categorical variables where any expected cell count was <5, Fisher’s exact test was performed; such cases are indicated by a superscript “#”.

### Screening of predictor variables using LASSO regression

In the training set, we used whether the patient was unplanned readmitted as the outcome variable and included a total of 36 candidate predictors. For variable selection, LASSO regression with the penalty parameter tuned via the one-standard-error rule (λ_1se = 0.029) identified and retained six predictors for the final model. These predictors were: smoking, PVD in T2DM, nutritional risk, duration of diabetes, HbA1c, and NLR. All six predictors exhibited high stability probabilities above 0.90 (range 0.927–0.998) across 1,000 bootstrap resamples, indicating robust variable selection. Although occupation, diabetic nephropathy, and diabetic retinopathy showed significant univariate associations (Table 1), they were not retained by LASSO; the coefficient path plot (Figure 2B) illustrates how the coefficients of these non-selected variables shrink to zero. The variable selection process is illustrated in Figures 2A,B.

**Figure 2:**
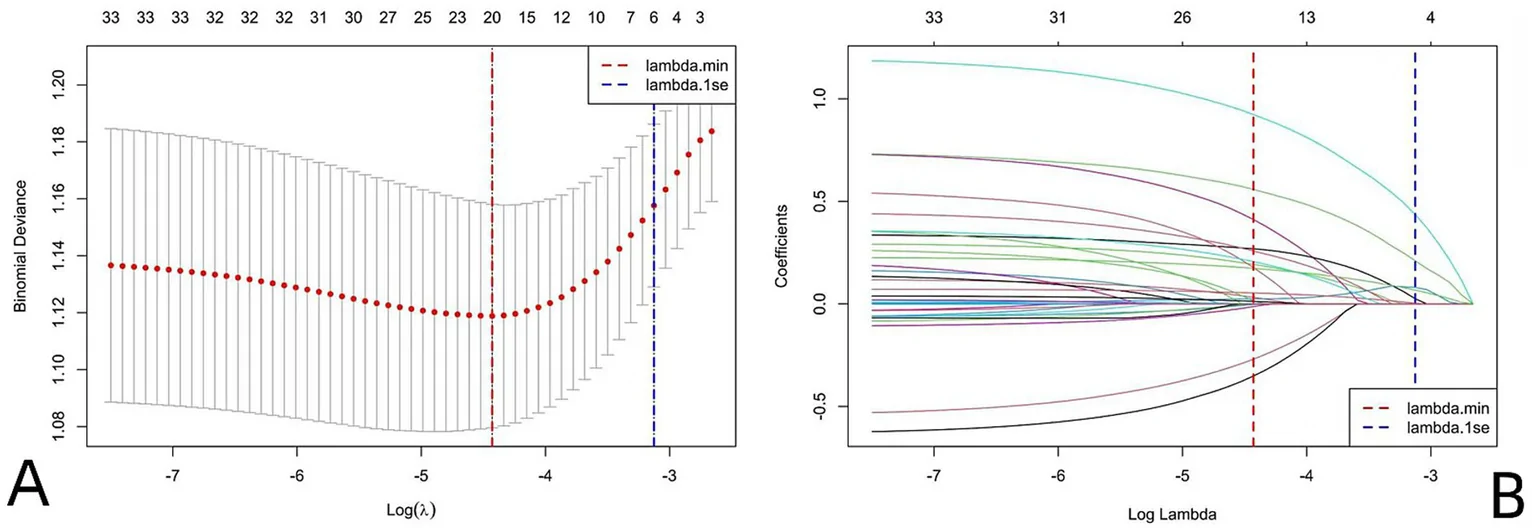
LASSO regression analysis for the selection of predictor variables. (A) The variation curve of the mean square error (mean ± standard error) of the ten-fold cross-validation with log(λ). The red dotted line represents λ_min_ (0.012), and the blue dotted line shows the final model selection λ_1se_ (0.029). (B) Coefficient path diagrams of all 36 predictor variables. The vertical line at λ_1se_ identifies the final model containing six non-zero coefficient predictor variables.

### Construction of an unplanned readmission prediction model

Based on the variables screened out by LASSO regression, we constructed a Logistic regression model for predicting unplanned readmission in patients with type 2 diabetes and drew a nomogram. The results of multivariate analysis showed that smoking, PVD in T2DM, nutritional risk, a longer course of diabetes, higher glycated hemoglobin levels, and NLR were independent risk factors for unplanned readmission in patients. Shown in Table 2 and Figure 3. The final logistic regression equation (refitted after LASSO) is: Logit(P) = −3.506 + 0.894 × smoking + 0.643 × PVD in T2DM + 0.045 × diabetes duration + 0.079 × HbA1c + 0.583 × nutritional risk + 0.166 × NLR.

**Table 2:** Risk factors for unplanned readmission in adult patients with type 2 diabetes.

Variables	*β*	S.E.	*Wald χ^2^*	*p*	OR	95% C.I. for OR	
						Lower	Upper
Smoking	0.894	0.272	10.826	0.001	2.445	1.435	4.163
PVD in T2DM	0.643	0.185	12.032	0.001	1.903	1.323	2.737
Nutritional risk	0.583	0.212	7.580	0.006	1.792	1.183	2.714
Duration of diabetes	0.045	0.011	17.105	0.000	1.046	1.024	1.069
HbA1c	0.079	0.032	6.276	0.012	1.083	1.017	1.152
NLR	0.166	0.050	11.257	0.001	1.181	1.072	1.302

**Figure 3:**
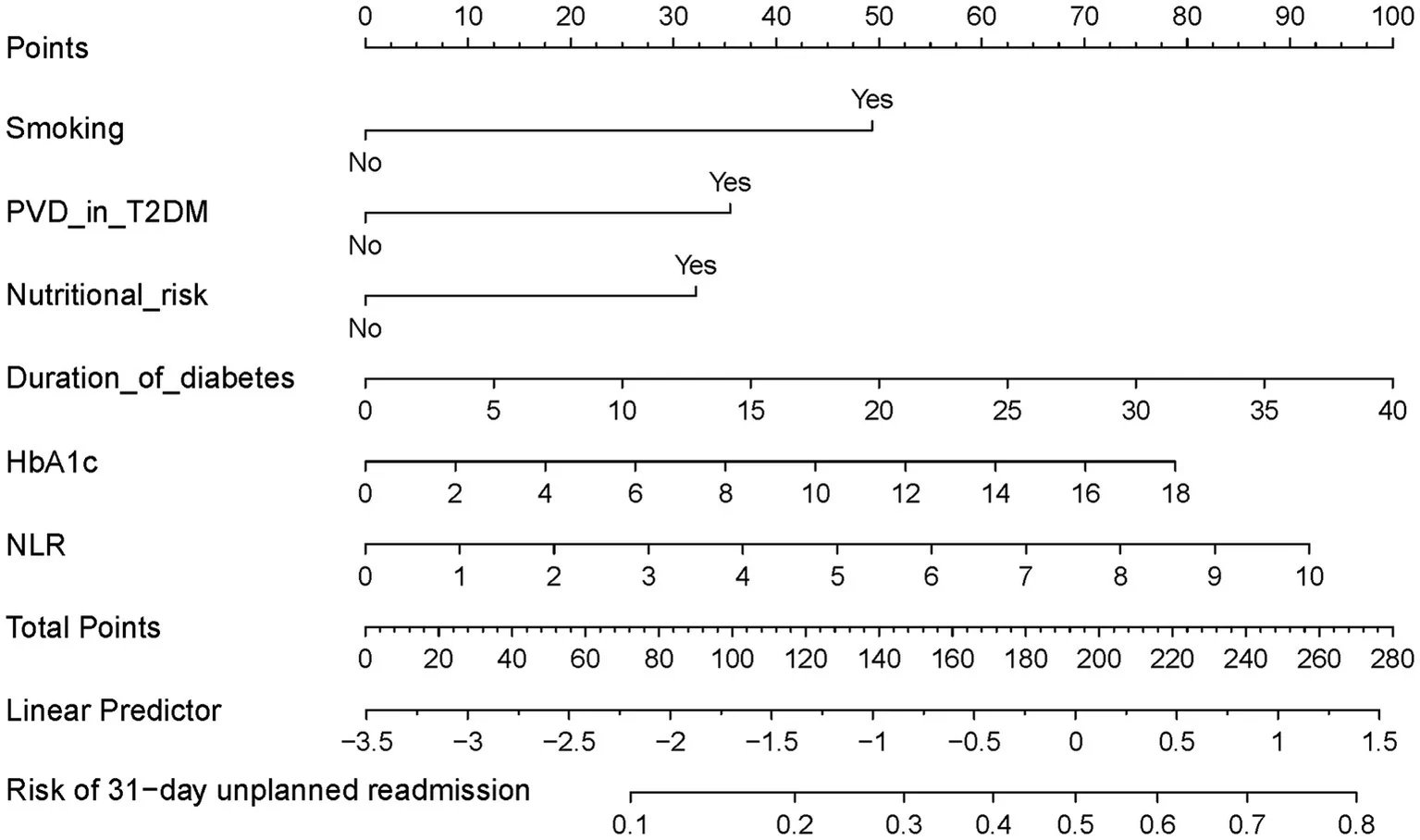
Nomogram for predicting the risk of 31‑day unplanned readmission in patients with type 2 diabetes. To use the nomogram, locate the value of each predictor on its corresponding axis, draw a vertical line to the “Points” axis to obtain the points for that predictor, sum all points to obtain the total points, and then draw a vertical line from the total points to the “Risk” axis to obtain the predicted probability. Clinical example: A patient with nutritional risk (NRS 2002 ≥ 3), diabetes duration of 15 years, HbA1c of 8.0%, and NLR of 2.5 (non‑smoker, without PVD) would have a total point score of approximately 125, corresponding to a predicted 31‑day unplanned readmission risk of about 23%.

### Verification of the nomogram model

The AUC of the model in the training set (Figure 4A) and the internal hold-out set (Figure 4B) was 0.846 (95% CI, 0.814–0.878) and 0.817 (95% CI, 0.761–0.873), respectively, indicating good discrimination. Using Harrell’s bootstrap optimism correction (1,000 iterations) with full repetition of the modeling process (LASSO selection + logistic refitting) in each bootstrap sample, the mean optimism was 0.016, yielding an optimism-corrected AUC of 0.830. Calibration was assessed using flexible calibration curves with 95% confidence intervals (Figures 5A,B): bootstrap for the training set and loess for the internal hold-out set. The training set showed calibration (intercept ≈ 0, slope = 1.00, Brier = 0.100). In the internal hold-out set, the calibration intercept was 0.122, slope was 1.002 (95% CI, 0.76–1.24), and Brier score was 0.113. The Hosmer-Lemeshow test *p* values were 0.709 (training set) and 0.652 (internal hold-out set); however, this test has limited power with *n* = 488 (internal hold-out set) and is reported only as a supplementary reference. Although the calibration plot showed slight overprediction in the higher risk deciles, the clinical significance of this overestimation remains uncertain, and its impact on decision-making should be assessed in prospective implementation studies. Decision curve analysis showed net clinical benefit within the clinically relevant threshold probability range of 0.05–0.40 (Figures 6A,B). To further illustrate clinical utility, we evaluated the nomogram’s performance at risk thresholds of 10, 15, and 20% using the internal hold-out set. The sensitivity, specificity, positive predictive value (PPV), and negative predictive value (NPV) are shown in Table 3. The 10, 15, and 20% thresholds presented here are illustrative examples to contextualize model sensitivity and specificity; they are not validated clinical decision thresholds and should not be used as such without external and prospective validation.

**Figure 4:**
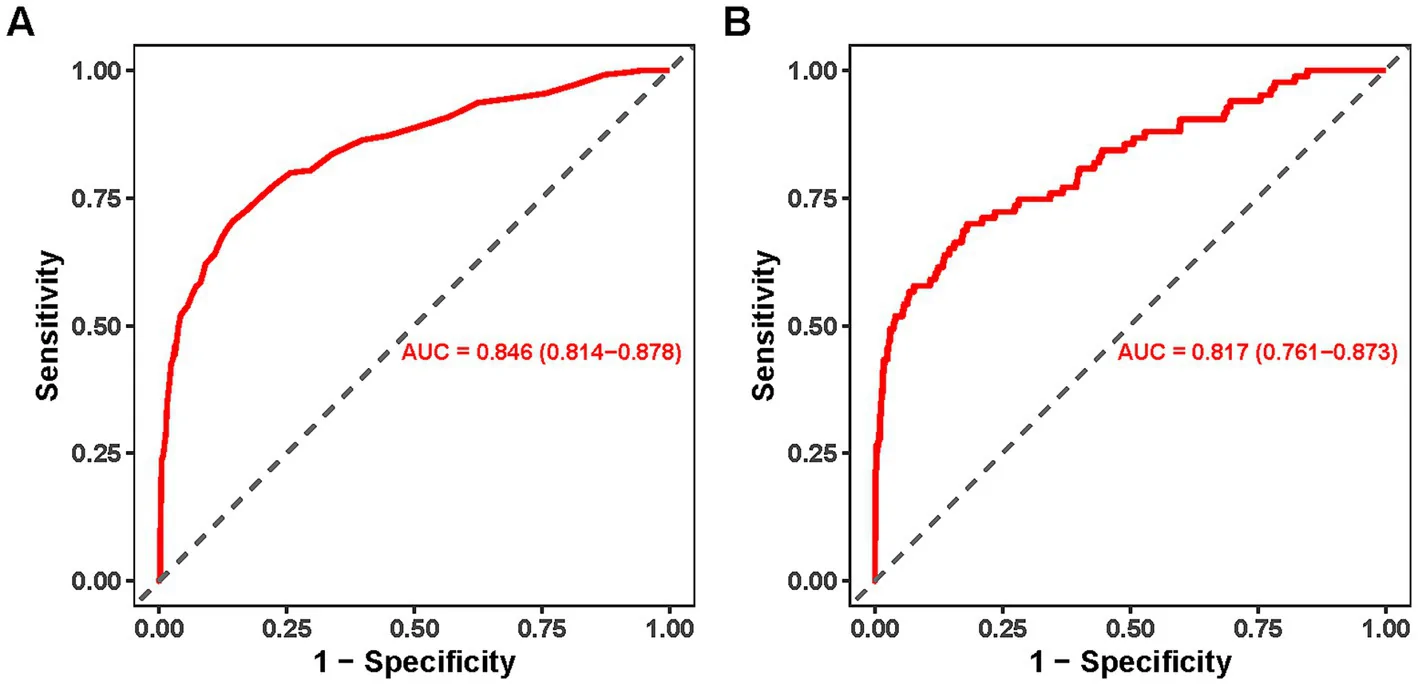
The AUC-ROC curves of predictive nomogram in **(A)** the training dataset and **(B)** the internal hold‑out dataset.

**Figure 5:**
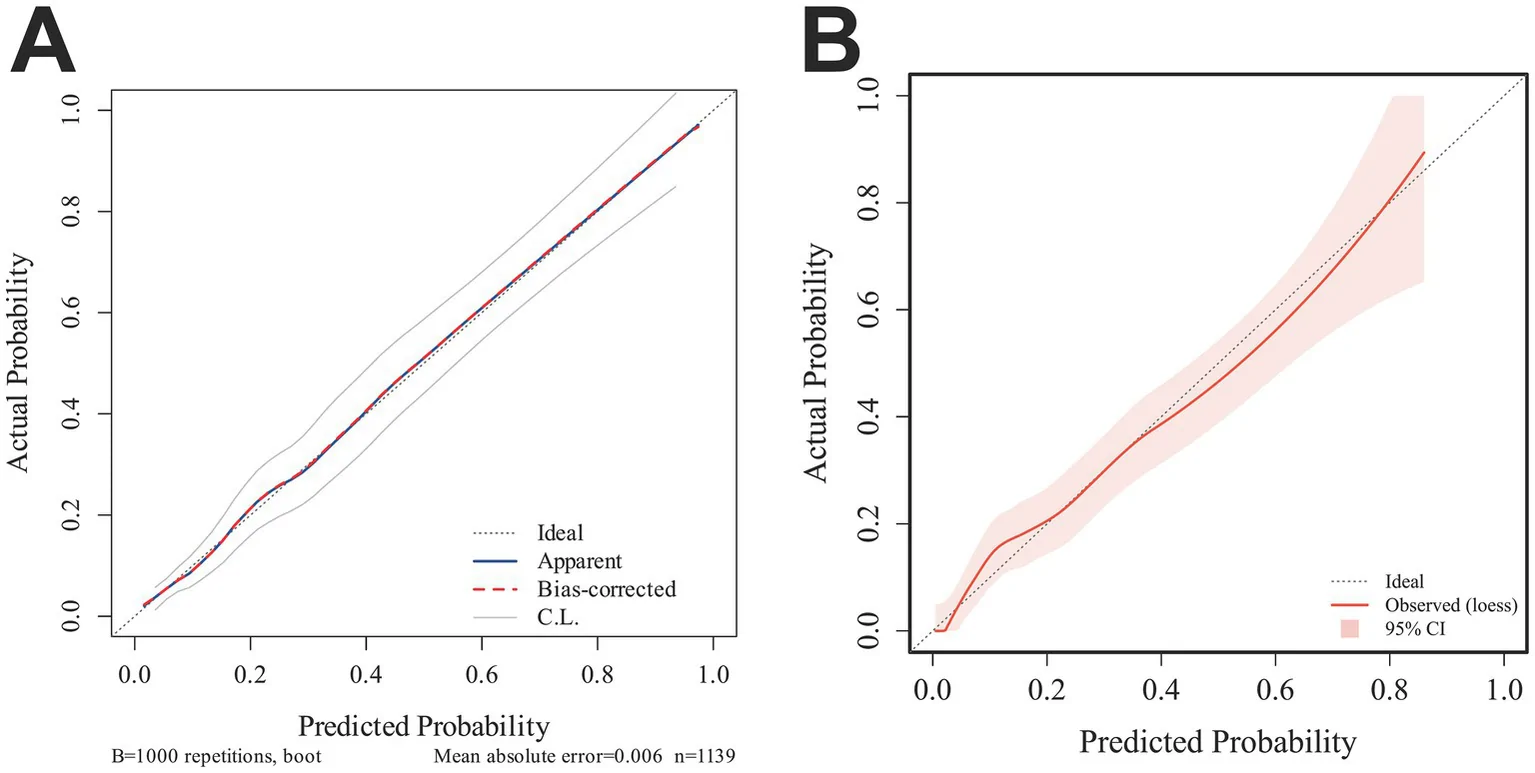
Calibration curves for **(A)** the training set and **(B)** the internal hold‑out set. **(A)** Training set: Calibration curves from bootstrap internal validation (B = 1,000). The “Apparent” curve shows the model’s performance on the training data, the “Bias-corrected” curve is the optimism-corrected estimate, and the shaded area (C.L.) represents the 95% confidence interval. The dashed line indicates ideal calibration. **(B)** Internal hold‑out set: Flexible loess‑smoothed calibration curve (red solid line) with pointwise 95% confidence intervals (light red shaded area). The dashed line indicates ideal calibration.

**Figure 6:**
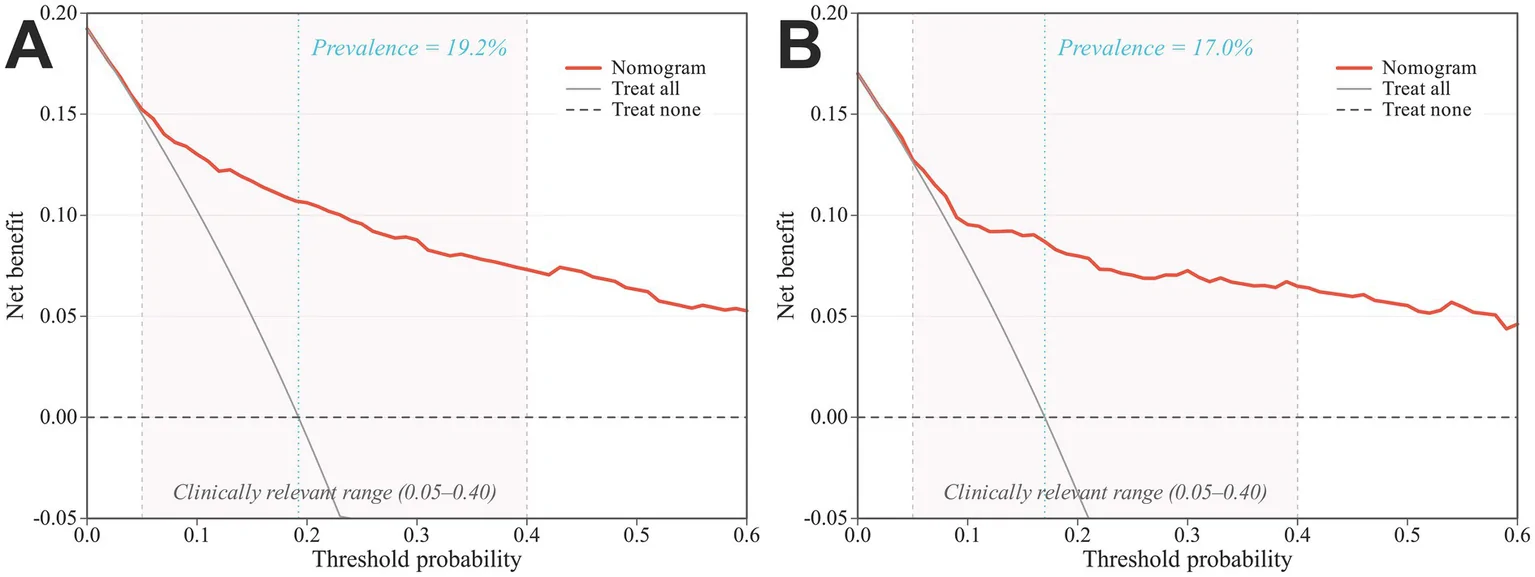
Decision curve analysis for (A) the training set and (B) the internal hold-out set. The shaded area denotes the clinically relevant threshold range (0.05–0.40). Vertical dotted lines indicate the event prevalence (training: 19.2%; hold-out: 17.0%). The horizontal dashed line represents a net benefit of zero.

**Table 3:** Performance of the nomogram at selected risk thresholds in the internal hold-out set.

Threshold	Sensitivity (%)	Specificity (%)	PPV (%)	NPV (%)
10.00%	91.57	31.36	21.47	94.78
15.00%	77.11	62.22	29.49	92.99
20.00%	68.67	81.98	43.85	92.74

## Discussion

In this study, the 31-day unplanned readmission rate among 1,627 diabetic patients was 18.50%. Our 31-day window (Chinese national standard) differs by only 1 day from the conventional 30-day definition, which does not impair comparability. This rate is higher than the 11.00% reported by Soh et al. (18) and 13.68% by Tan et al. (19), but lower than the 36.00% reported by Png et al. (20). This difference may be related to the different research methods, study populations, and readmission time Settings of each study. The unplanned readmission rate among diabetic patients is relatively high, which not only imposes a disease burden on the patients but also increases the pressure on the healthcare system. Therefore, identifying high-risk patients is crucial for preventing unplanned readmission and related adverse consequences.

Previous literature has shown (21) that diabetic complications are the most common influencing factor for readmission. Raval et al. (22) demonstrated that diabetic patients with complications had higher scores of the Deyo-Charlson comorbidity Index (D-CCI) and the Diabetes Complication Severity Index (DCSI), and a higher readmission rate. The results of this study show that among several complications, PVD is an influencing factor for readmission. PVD as a direct manifestation of macrovascular complications of diabetes, is not only the pathological basis for causing diabetic foot (such as ulcers, infections, and gangrene), a major cause of disability and long-term hospitalization, but also a sign of systemic atherosclerosis, significantly increasing the risk of hospitalization due to concurrent heart failure, acute coronary syndrome and other cardiovascular and cerebrovascular events (23). Modifiable risk factors, such as smoking, can accelerate the progression of peripheral vascular disease. Long-term smoking induces vascular endothelial damage, promotes systemic inflammation and oxidative stress. These effects, in synergy with hyperglycemia, intensify insulin resistance, blood glucose fluctuations, and accelerate the occurrence and progression of PVD (24). Therefore, in clinical practice, it is important to incorporate early screening for PVD and strong smoking cessation intervention into the standard care of diabetic patients.

This study found that a longer course of diabetes and a higher level of HbA1c are independent risk factors for unplanned readmission, which is consistent with the report by Regassa et al. (10). Their research shows that for each additional year of diabetes course, the risk of readmission increases by 14%, and a persistent hyperglycemic state also significantly raises the readmission rate. However, these two factors are not independent of each other but have a close pathophysiological relationship. On the one hand, as the course of diabetes prolongs, the pathological process of progressive failure of pancreatic β-cell function and intensified insulin resistance in patients deepens continuously, which itself leads to a significant increase in the difficulty of blood sugar control. Meanwhile, chronic complications of diabetes caused by a long course of the disease (such as kidney disease and autonomic neuropathy) will further interfere with the metabolism of hypoglycemic drugs and cause sharp fluctuations in blood sugar, making it more difficult to achieve the ideal blood sugar control target (25, 26). On the other hand, the persistent state of poor blood sugar control will accelerate the apoptosis of β cells and promote the occurrence and development of various complications, thus accelerating the deterioration of the natural course of the disease (27).

This study incorporated nutritional risk into the analysis and found it to be an independent influencing factor for readmission in patients with diabetes. The high prevalence of nutritional risk in diabetic patients has gained increasing recognition. A recent review reported that among hospitalized patients with type 2 diabetes, the prevalence of nutritional risk was 38% and the prevalence of malnutrition was 25%, both significantly associated with complication rates, mortality, and length of hospital stay (28). A study involving 21,807 elderly patients found that, after adjusting for confounding factors such as age, sex, and diagnosis, patients with nutritional risk at admission had a 1.44-fold higher risk of readmission within 30 days and a 1.47-fold higher risk within 180 days compared with those without nutritional risk (29). Furthermore, a prospective study by Sharma et al. (30) explicitly indicated that nutritional status at admission is an important predictor of readmission or death within 6 months after discharge, and that incorporating nutritional status into risk prediction models is necessary. Current readmission risk models for diabetic patients tend to focus on clinical and laboratory parameters such as HbA1c, diabetes duration, and comorbidities, while the contribution of nutritional status has received little attention. For clinical practice, implementing systematic nutritional screening at admission and developing targeted nutritional support strategies for high-risk patients may reduce unplanned readmission rates and improve the overall prognosis of this population.

Systemic chronic inflammation is one of the core pathological mechanisms for the occurrence, development and poor prognosis of diabetes. This study shows that NLR, as a complex inflammatory indicator, is an independent risk factor for readmission in diabetic patients. Hashmi et al. reported (31) that the levels of proinflammatory cytokines in diabetic patients were significantly higher than those in healthy groups, and the levels of proinflammatory cytokines such as TNF-α and IL-6 were positively correlated with poor blood glucose control. NLR can comprehensively reflect the activation of such inflammatory states. In addition, Leucuța et al. have shown (32) that NLR is a potential indicator for predicting complications and prognosis in patients with diabetes. The results of this study suggest that NLR may also have utility in readmission risk prediction, complementing its established role in predicting complications and prognosis in patients with diabetes. Additionally, although emergency department admission has been associated with readmission in some studies (33), we did not observe this trend in our cohort, possibly due to the small number of patients admitted via this route.

The LASSO regression method introduces a penalty coefficient in model estimation, which can effectively handle overfitting and multicollinearity (34). In this study, we used LASSO-logistic regression to screen and model the potential variables influencing 31-day unplanned readmission in patients with type 2 diabetes, and established a nomogram prediction model. The AUC of the training set and internal hold-out set were 0.846 and 0.817, respectively, indicating good and stable discrimination. Meanwhile, the calibration curve showed that the predicted probability was highly consistent with the observed outcomes, and decision curve analysis (DCA) further confirmed that the model provides significant clinical net benefit across a large threshold range. Based on LASSO regression, our nomogram incorporates nutritional, immune, and endocrine indicators into a nomogram, which may help to assess readmission risk in diabetic patients more comprehensively.

To translate this multi-dimensional nomogram into clinical practice, we evaluated its performance at three specific risk thresholds (10, 15, and 20%) using the internal hold-out set. A threshold of 10% provides high sensitivity (91.6%) and negative predictive value (94.8%), suggesting that patients below this threshold might represent a low-risk group in whom a less intensive discharge strategy could be considered in future prospective evaluation. A threshold of 20% offers better specificity (81.98%) and positive predictive value (43.85%), which is a meaningful improvement over the baseline readmission rate of 18.5% and may be preferred when resources are limited. The 15% threshold represents an intermediate option. Clinicians can choose the threshold according to their practice setting and the balance between sensitivity and specificity. Before clinical adoption, this model requires external validation in independent cohorts, ideally from multiple centers. The proposed risk thresholds should then be tested in a prospective interventional trial (e.g., comparing standard discharge vs. model-guided early discharge) to determine whether a threshold-based strategy actually improves patient outcomes without compromising safety.

### Limitations

This study has several limitations. First, it was a single-center study with only internal validation (no external validation), which limits the generalizability of our findings. External validation is needed before clinical use. Second, we did not collect information on physical activity or psychological status, which may be associated with readmission (2). Third, the candidate-stage EPV was approximately 6 (219 events/36 predictors), below the recommended minimum of 10; however, we used LASSO regression with cross-validation to mitigate overfitting, and the final model contained only six predictors (EPV ≈ 36) with stable performance (AUC 0.846 vs. 0.817). Fourth, we assumed linear relationships for the continuous predictors; post-hoc restricted cubic spline analyses revealed significant non-linear effects for HbA1c and NLR (both *P* < 0.05), whereas the linear assumption was supported for diabetes duration (*p* > 0.05). Consequently, our model may not fully capture the complexity of these relationships, and future studies should consider flexible modeling (e.g., splines); Fifth, although the proportion of missing data was low (4.3%) and missingness was assumed to be at random, complete-case analysis may introduce selection bias if this assumption was violated. However, comparison of baseline characteristics between included and excluded patients (Supplementary Table S2) showed no significant differences in observed variables. Nevertheless, this does not formally test the missing-at-random assumption, and the potential impact of non-random missingness on model performance cannot be excluded.

## Conclusion

This single-center retrospective study developed a nomogram incorporating nutritional risk to predict 31-day unplanned readmission in type 2 diabetes. The model showed acceptable internal discrimination and calibration. However, because the data came from a single hospital during one calendar year, the generalizability of these findings is uncertain. External validation in independent, geographically diverse, and temporally separate cohorts is needed before clinical application. Nevertheless, these results suggest that including nutritional risk may enhance readmission prediction and warrant further investigation.

## Data Availability

The dataset contains patient-level clinical information extracted from electronic medical records. Due to privacy and ethical restrictions (including the principles of the Declaration of Helsinki and local regulations), the data cannot be made publicly available. Access may be granted to qualified researchers upon reasonable request to the corresponding author and with approval from the institutional ethics committee. Requests to access the datasets should be directed to HX; JC Email: 2018050642@usc.edu.cn; 2018050781@usc.edu.cn.
