# Supplementary material for: A nomogram incorporating nutritional risk for predicting 31-day unplanned readmission in adults with type 2 diabetes mellitus using LASSO regression
Source: Front Nutr. 2026 Sep 30;13:1863130. doi: 10.3389/fnut.2026.1863130 (PMC13661480; doi:10.3389/fnut.2026.1863130)
Supplement: Supplementary file 1 [file Supplementary_file_1.zip › Supplementary Table 7.docx]

| **Supplementary Table S7. Candidate predictors for LASSO regression: variable types, units, and missing data**  **handling.** | | | |
| --- | --- | --- | --- |
| **Variable** | **Type** | **Unit** | **Missing handling** |
| Age | continuous | year | complete-case |
| Residences | categorical | — | complete-case |
| Education | categorical | — | complete-case |
| Occupation | categorical | — | complete-case |
| Smoking | categorical | — | complete-case |
| Drinking | categorical | — | complete-case |
| Gender | categorical | — | complete-case |
| Marital status | categorical | — | complete-case |
| Insurance type | categorical | — | complete-case |
| Admission route | categorical | — | complete-case |
| Discharge Method | categorical | — | complete-case |
| Length of hospital stay | continuous | day | complete-case |
| Clinical pathway | categorical | — | complete-case |
| Diabetic nephropathy in patient with type 2 diabetes | categorical | — | complete-case |
| Diabetic retinopathy in patient with type 2 diabetes | categorical | — | complete-case |
| Peripheral vascular disease in patient with type 2 diabetes | categorical | — | complete-case |
| Nutritional risk | categorical | — | complete-case |
| Hypertension | categorical | — | complete-case |
| Coronary heart disease | categorical | — | complete-case |
| Atrial fibrillation | categorical | — | complete-case |
| Cerebral infarction | categorical | — | complete-case |
| Chronic obstructive pulmonary disease | categorical | — | complete-case |
| Hyperlipidemia | categorical | — | complete-case |
| Hypothyroidism | categorical | — | complete-case |
| Operationed | categorical | — | complete-case |

| Duration of diabetes | continuous | year | complete-case |
| --- | --- | --- | --- |
| Lower extremity venous thrombosis | categorical | — | complete-case |
| HbA1c | continuous | % | complete-case |
| FBG, | continuous | mmol/L | complete-case |
| DD | continuous | mg/L | complete-case |
| UA | continuous | μmol/L | complete-case |
| LDL | continuous | mmol/L | complete-case |
| TG | continuous | mmol/L | complete-case |
| HDL | continuous | mmol/L | complete-case |
| TC | continuous | mmol/L | complete-case |
| NLR | continuous | — | complete-case |
