# Supplementary material for: A nomogram incorporating nutritional risk for predicting 31-day unplanned readmission in adults with type 2 diabetes mellitus using LASSO regression
Source: Front Nutr. 2026 Sep 30;13:1863130. doi: 10.3389/fnut.2026.1863130 (PMC13661480; doi:10.3389/fnut.2026.1863130)
Supplement: Supplementary file 1 [file Supplementary_file_1.zip › Supplementary Tables 1, 3, 4, 5, and 6.docx]

| Supplementary Table S1 | |
| --- | --- |
| Project | AUC/Bias |
| Original AUC | 0.846 |
| Bootstrap average AUC | 0.845 |
| Optimistic Bias | -0.001 |
| Corrected AUC | 0.847 |

| Supplementary Table S3. Variance inflation factors (VIFs) for the six predictors in the final logistic regression model. | |
| --- | --- |
| Variables | VIF |
| Smoking | 1.00 |
| Peripheral vascular disease in patient with type 2 diabetes | 1.09 |
| Nutritional risk | 1.09 |
| Duration of diabetes | 1.01 |
| Glycated hemoglobin | 1.01 |
| Neutrophil-to-Lymphocyte Ratio | 1.00 |

| Supplementary Table S4. Comparison of Wald and bootstrap 95% confidence intervals for odds ratios. | | |
| --- | --- | --- |
| Variables | 95% Wald CI | 95% Bootstrap CI |
| Smoking | 1.435 – 4.163 | 1.381 – 4.326 |
| Peripheral vascular disease in patient with type 2 diabetes | 1.323 – 2.737 | 1.241 – 2.868 |
| Nutritional risk | 1.183 – 2.714 | 1.124 – 2.809 |
| Duration of diabetes | 1.024 – 1.069 | 1.026 – 1.071 |
| Glycated hemoglobin | 1.017 – 1.152 | 1.025 – 1.146 |
| Neutrophil-to-Lymphocyte Ratio | 1.072 – 1.302 | 1.091 – 1.282 |

| Supplementary Table S5. Calibration performance measures for the training and internal hold‑out sets. | | | |
| --- | --- | --- | --- |
| Dataset | Calibration Intercept(95% CI) | Calibration Slope (95% CI) | Brier Score |
| Training (n=1139) | 0(1.28×10⁻¹¹) | 1.00 (0.98–1.02) | 0.1 |
| Internal hold‑out (n=488) | 0.122 (-0.153-0.397) | 1.002 (0.76–1.24) | 0.113 |

| **Supplementary Table S6. Key methodological and performance characteristics of the present model compared with selected existing diabetes readmission prediction models.** | | | | |
| --- | --- | --- | --- | --- |
| Feature / Model | Our Study | DERRI | Soh 2022 | Tan 2025 |
| Outcome | 31-day unplanned readmission | 30-day all-cause readmission | 30-day unplanned readmission | 30-day readmission in elderly T2DM |
| Sample Size | 1,627 (training cohort=1139，internal hold‑out set=488) | ~105,974 discharges (external validation cohort) | 2,355 | 8,800 |
| AUROC (AUC) | 0.846 (training) / 0.817 (internal hold‑out set) | ~0.634 – 0.643 | 0.68 (95% CI: 0.66-0.70) | 0.766 (95% CI: 0.745-0.787) |
| External Validation | No | Yes (external) | Yes (simulated) | Multicenter development |
| Key Predictors | Nutritional risk + Smoking history + PVD + disease duration + HbA1c + NLR | Admission labs, sociodemographics, comorbidities, prior discharge info | Length of stay, ischemic heart disease, PVD, number of drugs | Albumin (most significant), among 38 variables in derivation model |
| Nutritional Dimension | Systematically incorporated | Absent | — | — |
