# Supplementary material for: A nomogram incorporating nutritional risk for predicting 31-day unplanned readmission in adults with type 2 diabetes mellitus using LASSO regression
Source: Front Nutr. 2026 Sep 30;13:1863130. doi: 10.3389/fnut.2026.1863130 (PMC13661480; doi:10.3389/fnut.2026.1863130)
Supplement: Supplementary file 1 [file Supplementary_file_1.zip › Supplementary Table 2.docx]

| Supplementary Table S2. Comparison of baseline characteristics between the final analytic cohort (n = 1,627) and patients excluded due to missing data (n = 78). | | | | |
| --- | --- | --- | --- | --- |
| Variable | Group excluded due to missing data (n=78) | Included in the analysis group (n=1627) | t | P |
| Age,year(mean±SD) | 65.22±13.54 | 63.06±10.82 | 1.695 | 0.090 |
| Gender(male),n(%) | 48(61.54) | 925(56.85) | 0.667 | 0.414 |
| Duration of diabetes, year,Median (p25,p75) | 10.15(5.83,14.3) | 11.40(6.50,16.50) | -1.87 | 0.062 |
| HbA1c,%,(mean±SD) | 7.37±2.88 | 7.79±2.42 | -1.47 | 0.142 |
| Hypertension,n(%) | 40(51.28) | 1008(61.95) | 3.579 | 0.059 |
